# Advanced reperfusion therapies for acute pulmonary embolism in pregnancy and postpartum

**DOI:** 10.1093/ehjacc/zuag047

**Published:** 2026-03-31

**Authors:** Ioannis T Farmakis, Konstantinos C Christodoulou, Stefano Barco, Karsten Keller, Stavros V Konstantinides, Lukas Hobohm

**Affiliations:** Center for Thrombosis and Hemostasis, University Medical Center of the Johannes Gutenberg University, Langenbeckstr. 1, Mainz 55131, Germany; Department of Medicine I, LMU University Hospital, LMU Munich, Germany; Department of Cardiac and Vascular Surgery, University Medical Centre Mainz, Johannes Gutenberg University, Mainz, Germany; Department of Angiology, University Hospital Zurich, Zurich, Switzerland; Center for Thrombosis and Hemostasis, University Medical Center of the Johannes Gutenberg University, Langenbeckstr. 1, Mainz 55131, Germany; Department of Cardiology, University Medical Center Mainz (Johannes Gutenberg-University Mainz), Mainz 55131, Germany; Center for Thrombosis and Hemostasis, University Medical Center of the Johannes Gutenberg University, Langenbeckstr. 1, Mainz 55131, Germany; Department of Cardiology, Democritus University of Thrace, Alexandroupolis, Greece; Center for Thrombosis and Hemostasis, University Medical Center of the Johannes Gutenberg University, Langenbeckstr. 1, Mainz 55131, Germany; Department of Cardiology, University Medical Center Mainz (Johannes Gutenberg-University Mainz), Mainz 55131, Germany

**Keywords:** Pulmonary embolism, Systemic thrombolysis, Catheter-directed therapy, Pregnancy, Maternal mortality

## Abstract

**Aims:**

Pulmonary embolism (PE) remains the leading preventable cause of maternal death in high-income countries. Data on the management of acute PE during pregnancy are limited because pregnant women are systematically excluded from interventional trials. We evaluated the use and outcomes of advanced reperfusion therapies in pregnancy-associated PE.

**Methods:**

We performed a cross-sectional analysis of the US Nationwide Inpatient Sample from 2016 through 2020. Pregnancy- and puerperium-related hospitalizations were identified using the International Classification of Diseases, Tenth Revision, Clinical Modification (ICD-10-CM) codes. High-risk PE was defined by shock, mechanical ventilation, cardiac arrest, cardiopulmonary resuscitation, or vasopressor support. Advanced reperfusion therapies included systemic thrombolysis (ST), catheter-directed therapy (CDT), surgical embolectomy, and venoarterial extracorporeal membrane oxygenation (VA-ECMO).

**Results:**

Among 19 741 880 pregnancy- or puerperium-related hospitalizations, 12 715 (0.06%) were complicated by PE, corresponding to 6.7 (95% CI, 6.6–6.8) hospitalizations per 10 000 live births. The overall in-hospital mortality rate was 0.86% (0.058 [95% CI, 0.048–0.070] deaths per 10 000 live births). Mortality was 0.2% among patients without haemodynamic instability and 29.1% among those with high-risk PE. Advanced reperfusion therapies were used in 3.4% of all pregnancy-associated PE cases and in 32.7% of high-risk cases. In-hospital mortality was 9.8% after ST, 10.7% after CDT, and 50% after VA-ECMO. Major bleeding occurred in 15.7%, 10.7%, and 50%, respectively.

**Conclusion:**

Pregnancy-associated PE is rare but associated with high mortality in high-risk presentations. Advanced reperfusion therapies are used infrequently, reflecting the limited evidence, highlighting the need for multidisciplinary management, and further research and guidance.

Key points

- Pregnancy-associated PE is rare but associated with markedly increased mortality in high-risk presentations.
- Use of advanced reperfusion therapies, especially systemic thrombolysis, remains infrequent, despite their potential life-saving role in haemodynamically unstable PE.
- Prevention and early recognition of VTE during pregnancy are crucial, highlighting the need for standardized thromboprophylaxis and multidisciplinary management strategies.

## Introduction

The burden of cardiovascular diseases in pregnancy has increased substantially over the past decades.^1^ Pulmonary embolism (PE) is the main preventable cause of maternal death in high-income countries.^2^ Pregnant and postpartum women have up to a sixfold higher risk of venous thromboembolism than nonpregnant women of reproductive age, with a case fatality rate of approximately 3.5%.^3^ Data from a 33-year Swedish registry, including nearly 2 million women, reported an incidence of first-time VTE of 150.8 per 100.000 deliveries and demonstrated a steady annual increase of pregnancy-related PE by 5%.^4^ A case-control study from the United Kingdom, using the UK Obstetric Surveillance System, showed that PE accounted for 1.26 deaths per 100,000 pregnancies.^5^

Information on management of acute PE, and especially regarding the use of advanced treatments, during pregnancy remains limited in the guidelines, as pregnant women are systematically excluded from most interventional trials.^6^ In general, rapid and effective anticoagulation, primarily low-molecular-weight heparin, is the treatment of choice for the management of PE in pregnant patients.^3^ Although pregnancy is considered a relative contraindication to systemic thrombolysis (ST), due to the associated bleeding risk and foetal complications, in high and in selected cases of intermediate-high risk PE, reperfusion treatments—namely, ST, catheter-directed treatments (CDT) or surgical embolectomy—are to be considered, as in every other PE patient with impending or overt haemodynamic decompensation.^7^ However, evidence regarding the optimal use and outcomes of these interventions in pregnancy is sparse, making clear guideline recommendations difficult.^8^ We, therefore, investigated the use of advanced therapies and their outcomes in pregnancy-associated PE using a large nationwide database.

## Methods

For this cross-sectional analysis, we used data (years 2016–2020) from the Nationwide Inpatient Sample (NIS), the largest publicly available, all-payer, inpatient healthcare database in the United States. The NIS is maintained by the Agency for Healthcare Research and Quality as part of the Healthcare Cost and Utilization Project (HCUP) and includes a 20% stratified sample of discharges from U.S. community hospitals, excluding rehabilitation and long-term acute care facilities. The database represents approximately 97% of the U.S. population and captures a comprehensive spectrum of inpatient encounters. Because the NIS contains only de-identified information, this study was exempt from institutional review board approval and informed consent.

We identified pregnancy- and puerperium-associated hospitalizations as the International Classification of Diseases, Tenth Revision, Clinical Modification (ICD-10-CM) O88.2 (obstetric thromboembolism), or the combination I26 (PE) plus O09 (supervision of high-risk pregnancy). Hospitalizations with O88.23 diagnosis were defined as occurring in the puerperium. High-risk hospitalizations were characterized by shock, ventilator support, cardiac arrest, cardiopulmonary resuscitation, or vasopressors. Advanced therapy was defined as ST, CDT (including catheter-directed thrombolysis and catheter-based thrombectomy), surgical embolectomy (SE), and veno-arterial extracorporeal membrane oxygenation (VA-ECMO). The number of pregnancy-associated diagnoses of PE and the number of fatalities caused by pregnancy-associated PE were divided by the number of live births (obtained from the US Census Bureau) to calculate crude incidence rates and crude fatality rates, respectively; the Wilson score interval with continuity correction was used to estimate 95% confidence intervals (CI). National estimates were obtained using discharge-level weights supplied in the datasets. R was used for statistical analysis.

## Results

We estimated 19 741 880 maternal-related hospitalizations and 1 956 190 PE-related hospitalizations between 2016–2020. Of these, 12 715 (0.06% of maternal-related hospitalizations and 0.64% of PE-related hospitalizations) were pregnancy-associated PE hospitalizations, corresponding to an incidence of 6.7 (95%CI, 6.6–6.8) per 10 000 live births. The median age (IQR) of the patients was 30 (26.0, 34.0) years. High-risk PE accounted for 2.2% of pregnancy-associated PE hospitalizations with substantially higher comorbidity rates. The baseline characteristics of the included sample are presented in *Table 1*. The median length of hospital stay was 3 (IQR 2.0–5.0) days for the overall and low-risk PE groups, and 8.5 (IQR 2.0–21.2) days for high-risk PE. The in-hospital case fatality rate was 0.86%, corresponding to a maternal PE mortality rate of 0.058 (95%CI, 0.048–0.070) deaths per 10 000 live births. In the absence of haemodynamic instability, fatality rate was 0.2%, while among high-risk PE, almost one third of the patients (29.1%) died during hospitalization.

**Table 1:** Baseline characteristics, management, and outcomes of maternal-associated pulmonary embolism hospitalizations according to risk status

Characteristic	Overall, N = 12,715^a^	Not high-risk, N = 12,440^a^	High-risk, N = 275^a^	*P*-value*
Age	30.0 (26.0, 34.0)	30.0 (26.0, 34.0)	31.0 (26.8, 35.0)	0.2
Race				0.6
White	5940 (49)	5795 (48)	145 (54)	
Black	3540 (29)	3480 (29)	60 (22)	
Hispanic	1850 (15)	1815 (15)	35 (13)	
Asian/Pacific Islander	340 (2.8)	325 (2.7)	15 (5.6)	
Native American	80 (0.7)	80 (0.7)	0 (0)	
Other	470 (3.8)	455 (3.8)	15 (5.6)	
Puerperium	6605 (52)	6465 (52)	140 (51)	0.9
*Comorbidities*				
Diabetes	395 (3.1)	380 (3.1)	15 (5.5)	0.3
Hypertension	320 (2.5)	300 (2.4)	20 (7.3)	0.023
Obesity	2800 (22)	2715 (22)	85 (31)	0.11
Heart failure	450 (3.5)	385 (3.1)	65 (24)	**<0**.**001**
Atrial fibrillation	50 (0.4)	35 (0.3)	15 (5.5)	**<0**.**001**
Ischemic heart disease	235 (1.8)	210 (1.7)	25 (9.1)	**<0**.**001**
Thrombophilia^b^	975 (7.7)	955 (7.7)	20 (7.3)	>0.9
Smoking	1165 (9.2)	1135 (9.1)	30 (11)	0.6
History of VTE	1115 (8.8)	1105 (8.9)	10 (3.6)	0.2
Post-partum infections	800 (6.3)	755 (6.1)	45 (16)	**0**.**002**
Hypertensive disorders of pregnancy	1465 (12)	1430 (11)	35 (13)	0.8
Gestational diabetes	395 (3.1)	380 (3.1)	15 (5.5)	0.3
Pre-eclampsia or eclampsia	925 (7.3)	895 (7.2)	30 (11)	0.3
*Management*				
Use of any advanced treatment	435 (3.4)	345 (2.8)	90 (33)	**<0**.**001**
Systemic thrombolysis	255 (2.0)	220 (1.8)	35 (13)	**<0**.**001**
Catheter-directed thrombolysis	90 (0.7)	65 (0.5)	25 (9.1)	**<0**.**001**
Catheter-based thrombectomy	75 (0.6)	60 (0.5)	15 (5.5)	**<0**.**001**
Surgical embolectomy	30 (0.2)	NR (0.2)	NR (3–4)	**<0**.**001**
VA-ECMO	30 (0.2)	0 (0)	30 (11)	**<0**.**001**
*Outcomes*				
Length of stay (days)	3.0 (2.0, 5.0)	3.0 (2.0, 5.0)	8.5 (2.0, 21.2)	**<0**.**001**
In-hospital mortality	110 (0.9)	30 (0.2)	80 (29)	**<0**.**001**
Abortive outcome	215 (1.7)	NR (1.6)	NR (3–4)	0.3
Bleeding	1635 (13)	1560 (13)	75 (27)	**0**.**001**
Caesarean section	2040 (16)	1945 (16)	95 (35)	**<0**.**001**
Pre-term delivery	435 (3.4)	NR (3.4)	NR (3–4)	>0.9
Total charges (US dollars)	26 253 (15,652, 50,026)	25 884 (15,479, 47,957)	177 468 (76,864, 381,488)	**<0**.**001**

Bold text highlights statistical significance.

PE, pulmonary embolism; NR, not reported due to HCUP privacy protection (Reporting cell sizes <10 increases the risk of re-identification and is discouraged, as specified in the Data Use Agreement); VA-ECMO, veno-arterial extracorporeal membrane oxygenation; VTE, venous thromboembolism.

^a^Median (IQR); *n* (%)

^b^Thrombophilia was defined through the use of ICD-10 codes D68.5 (Primary thrombophilia: Activated protein C resistance, Prothrombin gene mutation, or other primary thrombophilia) and D68.6 (Other thrombophilia: Antiphospholipid syndrome, Lupus anticoagulant syndrome, Other thrombophilia).

^*^Wilcoxon rank-sum test for complex survey samples; chi-squared test with Rao & Scott's second-order correction

Advanced reperfusion therapies were used in 3.4% of all pregnancy-associated PE cases (pregnancy, 2.2%; puerperium, 4.5%), and in 32.7% of high-risk PE (pregnancy: 37.0%, puerperium: 28.6%). Among both high-risk and non–high-risk PE, ST was the most frequently used advanced therapy (high-risk: 12.7%, non-high-risk: 1.8%), followed by CDT (high-risk: 14.5% non-high-risk: 1.0%); VA-ECMO was employed in 10.9% of high-risk patients. In-hospital mortality was 9.8% among those treated with ST and 10.7% among those receiving CDT; half (50%) of patients requiring VA-ECMO died. Major bleeding occurred in 15.7% of patients treated with ST, 10.7% of those treated with CDT, and 50% of those requiring VA-ECMO. Bleeding risk increased progressively with advancing maternal age, peaking in patients aged 40 years or older. Conversely, use of advanced therapies declined with age, whereas rates of in-hospital mortality remained low across all age groups, with only a modest increase in mortality in the oldest patients. Outcomes following advanced therapies are presented in the *Figure 1*.

**Figure 1:**
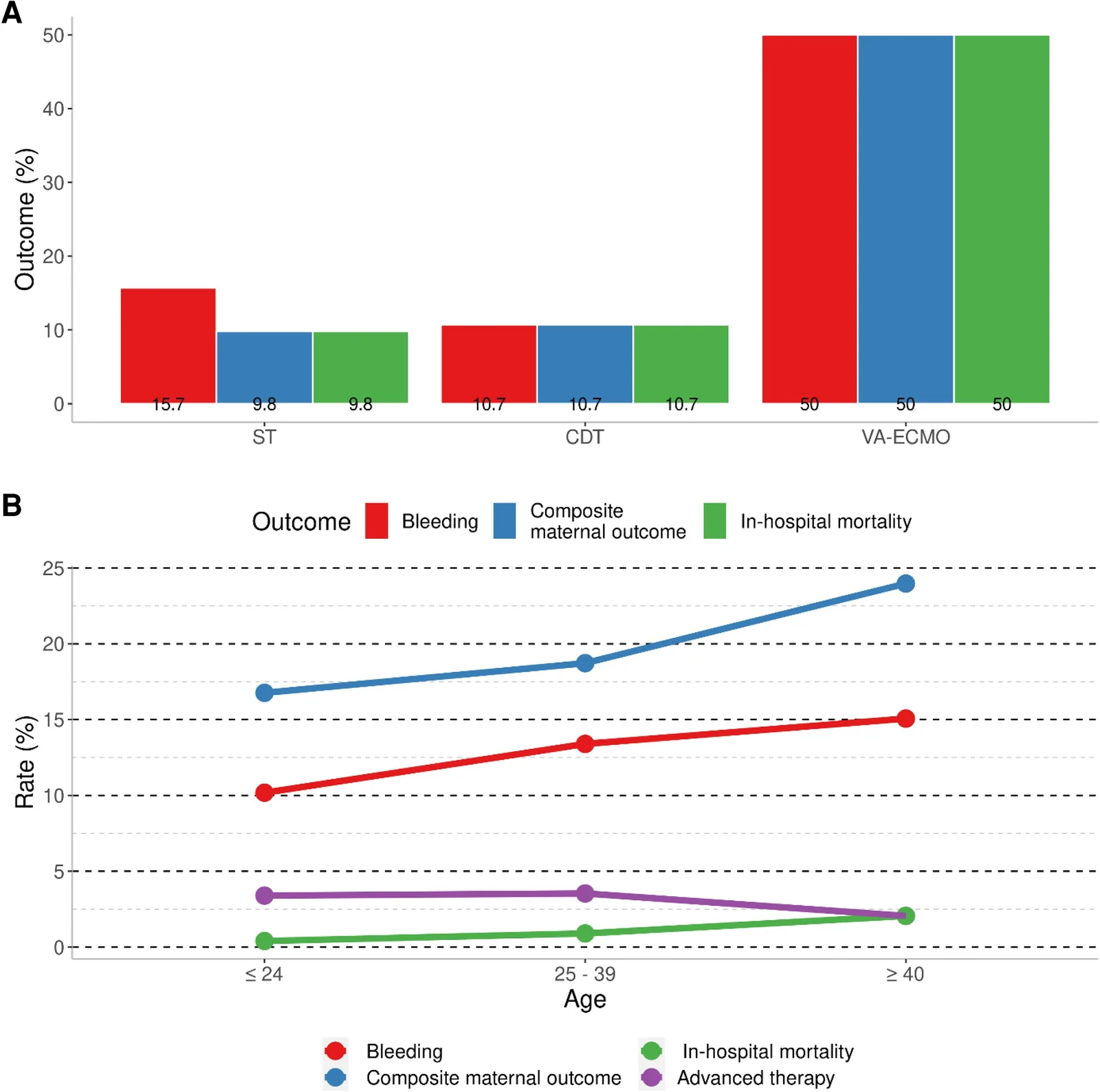
Outcomes of advanced reperfusion therapies and maternal age in pregnancy-associated pulmonary embolism. Panel *A*: Rates of major bleeding, composite maternal outcome, and in-hospital mortality among patients treated with systemic thrombolysis, catheter-directed therapy, and venoarterial extracorporeal membrane oxygenation. Panel *B*: Rates of bleeding, composite maternal outcome, in-hospital mortality, and use of advanced reperfusion therapy according to maternal age group. The composite maternal outcome consists of at least one of abortion, caesarean section, or pre-term delivery. ST, systemic thrombolysis; CDT, catheter-directed treatment; VA-ECMO, veno-arterial extracorporeal membrane oxygenation.

## Discussion

This nationwide analysis of pregnancy-associated PE hospitalizations indicates a very low overall fatality rate (0.2%) in the absence of haemodynamic instability, but a persistently high mortality (approximately 30%) among patients with high-risk PE. In the same context, a recent epidemiologic study using data from the US Centers for Disease Control and Prevention (CDC) showed that over the last 20 years, the PE-associated mortality rate was 0.1 per 10 000 live births.^2^ Despite clear indications for reperfusion in the general population of haemodynamically unstable PE, advanced therapies were infrequently used in this cohort, reflecting the sparse evidence and weak guideline recommendations for the use of advanced therapies (especially other than ST) in this population.^9,10^ Compared to previous nationwide data from Germany, the use of ST was considerably lower in the present US sample.^8^ On the other hand, we found a substantial (15%) use of CDT among patients with high-risk PE during pregnancy or the puerperium, with a fatality rate of 10.7%, aligning with recent data forecasting the increased penetration of CDT use.^11^ Of note, CDT had not been reported in the German sample dating back to an earlier period (2005–2016). In a systematic review including 127 pregnant women with PE, 6% (5 of 83) of those treated with ST died, whereas no deaths occurred among patients treated with CDT.^12^ Our results now indicate a higher (10%) fatality rate associated with CDT, likely pointing to a strong publication bias for these interventions in the pregnancy setting. Similarly, in a systematic review of 21 pregnancy-associated PE cases managed with VA-ECMO, mortality was 24%, which is half compared to the one we report here.^13^

Although the present analysis suffers from the NIS limitations, including reliance on administrative coding, lack of detailed clinical parameters, and inability to capture timing of interventions, it is hypothesis-generating regarding the safety and efficacy of reperfusion therapies in pregnancy-associated PE, and it underscores their persistently infrequent use in women with haemodynamic instability. The decision to pursue invasive treatment is likely influenced by concerns about maternal haemorrhage, foetal safety, and limited multidisciplinary experience. Enhanced awareness, standardized protocols, and, most importantly, specialized multidisciplinary management (through PERT) involving obstetrics, cardiology, and critical care should guide the employment of advanced therapy in life-threatening PE and may improve outcomes.^14^

Understanding the timing and circumstances of VTE during pregnancy is essential for developing and refining prevention and monitoring strategies to reduce maternal morbidity and mortality.^15^ Preventive strategies remain critical. Prospective, multinational cohort data are urgently needed to better characterize optimal prevention and management approaches in this vulnerable population.

## Data Availability

The data underlying this article are available in the article.
